# Access to HIV care in the context of universal test and treat: challenges within the ANRS 12249 TasP cluster-randomized trial in rural South Africa

**DOI:** 10.7448/IAS.19.1.20913

**Published:** 2016-06-01

**Authors:** Mélanie Plazy, Kamal El Farouki, Collins Iwuji, Nonhlanhla Okesola, Joanna Orne-Gliemann, Joseph Larmarange, France Lert, Marie-Louise Newell, François Dabis, Rosemary Dray-Spira, for the Anrs 12249 Tasp Study Group

**Affiliations:** 1 INSERM U1219 – Centre Inserm Bordeaux Population Health, Université de Bordeaux, Bordeaux, France; 2 Centre INSERM U1219 – Bordeaux Population Health, ISPED, Université de Bordeaux, Bordeaux, France; 3 INSERM, UMR_S 1136, Pierre Louis Institute of Epidemiology and Public Health, Team of research in Social Epidemiology, F-75013, Paris, France; 4 Sorbonne Universités, UPMC Univ Paris 06, UMR_S 1136, Pierre Louis Institute of Epidemiology and Public Health, Team of research in Social Epidemiology, F-75013, Paris, France; 5 Africa Centre for Population Health, University of KwaZulu-Natal, Mtubatuba, South Africa; 6 Research Department of Infection and Population Health, UCL, London, UK; 7 Centre Population et Développement, Ceped UMR 196 Paris Descartes IRD, IRD, Paris, France; 8 INSERM, U1018, Centre for Research in Epidemiology and Population Health, CESP, Epidemiology of Occupational and Social Determinants of Health, Villejuif, France; 9 Human Health and Development, Faculty of Medicine, University of Southampton, Southampton, UK; 10 Global Health Research Institute, University of Southampton, Southampton, UK

**Keywords:** HIV/AIDS, home-based HIV counselling and testing, linkage to care, universal test and treat, South Africa

## Abstract

**Introduction:**

We aimed to quantify and identify associated factors of linkage to HIV care following home-based HIV counselling and testing (HBHCT) in the ongoing ANRS 12249 treatment-as-prevention (TasP) cluster-randomized trial in rural KwaZulu-Natal, South Africa.

**Methods:**

Individuals ≥16 years were offered HBHCT; those who were identified HIV positive were referred to cluster-based TasP clinics and offered antiretroviral treatment (ART) immediately (five clusters) or according to national guidelines (five clusters). HIV care was also available in the local Department of Health (DoH) clinics. Linkage to HIV care was defined as TasP or DoH clinic attendance within three months of referral among adults not in HIV care at referral. Associated factors were identified using multivariable logistic regression adjusted for trial arm.

**Results:**

Overall, 1323 HIV-positive adults (72.9% women) not in HIV care at referral were included, of whom 36.9% (*n*=488) linked to care <3 months of referral (similar by sex). In adjusted analyses (*n*=1222), individuals who had never been in HIV care before referral were significantly less likely to link to care than those who had previously been in care (<33% vs. >42%, *p*<0.001). Linkage to care was lower in students (adjusted odds-ratio [aOR]=0.47; 95% confidence interval [CI] 0.24–0.92) than in employed adults, in adults who completed secondary school (aOR=0.68; CI 0.49–0.96) or at least some secondary school (aOR=0.59; CI 0.41–0.84) versus ≤ primary school, in those who lived at 1 to 2 km (aOR=0.58; CI 0.44–0.78) or 2–5 km from the nearest TasP clinic (aOR=0.57; CI 0.41–0.77) versus <1 km, and in those who were referred to clinic after ≥2 contacts (aOR=0.75; CI 0.58–0.97) versus those referred at the first contact. Linkage to care was higher in adults who reported knowing an HIV-positive family member (aOR=1.45; CI 1.12–1.86) versus not, and in those who said that they would take ART as soon as possible if they were diagnosed HIV positive (aOR=2.16; CI 1.13–4.10) versus not.

**Conclusions:**

Fewer than 40% of HIV-positive adults not in care at referral were linked to HIV care within three months of HBHCT in the TasP trial. Achieving universal test and treat coverage will require innovative interventions to support linkage to HIV care.

## Introduction

Initiating antiretroviral treatment (ART) as early as possible after acquiring HIV infection results in better health outcomes, reducing HIV-related morbidity or mortality [1,2]. Further, decreasing viral load with ART significantly reduces HIV transmission from the treated infected to the uninfected sexual partner in HIV-discordant couples [3–5]. Results from mathematical models suggest that universal and repeat HIV testing followed by immediate ART initiation could substantially decrease HIV incidence at population level [6]; this has been supported by subsequent results from an observational cohort study in rural KwaZulu-Natal, South Africa [7]. The World Health Organization (WHO) recently updated its HIV treatment and prevention guidelines, recommending universal test and treat with ART to be initiated in anyone living with HIV, regardless of clinical or immunological stage [8]. This was translated into the programmatic UNAIDS HIV “90–90–90” treatment targets, aiming for “90% of people living with HIV knowing their HIV status, 90% of those with diagnosed HIV infection receiving ART and 90% of those receiving ART having durable viral suppression” by 2020 [9].

South Africa carries one of the highest HIV burdens worldwide, with an estimated 6.3 million people living with HIV in 2013, and an HIV prevalence of 19.1% among 15- to 49-year-olds [10]. To achieve universal HIV testing in such a high HIV prevalence setting, community-based HIV testing services should be offered in addition to those offered in health facilities [11]. Among them, Home-Based HIV Counseling and Testing (HBHCT) has been shown to be acceptable and effective in increasing the number of people who know their HIV status, especially in South Africa [12–18], but there are limited data on linkage to care following HBHCT [19]. Further, while it is crucial that all HIV-identified individuals access HIV care and initiate ART as soon as possible, results from studies in sub-Saharan Africa have previously shown that many newly diagnosed HIV-positive people do not enter HIV care immediately following HIV diagnosis consequently delaying time to ART initiation [20,21].

The objectives of our analysis were to quantify the proportion of adults never or not currently in care who linked to HIV care within three months following an HIV diagnosis through HBHCT and to investigate factors associated with linkage to HIV care. The analysis was performed within the context of a cluster-randomized trial of treatment as prevention (TasP) conducted in rural and high HIV prevalence area in KwaZulu-Natal province, South Africa.

## Methods

### Study setting

We used data from the ANRS 12249 TasP trial, an ongoing cluster-randomized trial evaluating the effectiveness of immediate ART on HIV incidence. The trial is implemented since March 2012 in Hlabisa sub-district, northern KwaZulu-Natal, South Africa, a largely rural area, with scattered homesteads, an estimated HIV prevalence of 29% [22] and a decentralized local HIV programme [23].

### Trial procedures

The TasP trial protocol has been described previously [24,25]. HBHCT is offered every six months to eligible members of the trial communities contacted during home visits. Household members are informed about the trial objectives and procedures, and about ART eligibility criteria according to their cluster of residence. All participants identified as HIV positive receive a TasP referral card and are encouraged to access the TasP trial clinic in their cluster, situated <45 minutes walking distance from where they live.

In TasP trial clinics of the intervention clusters, ART initiation is offered immediately to all HIV-positive adults regardless of their CD4 count or clinical staging. In control clusters, ART initiation is offered according to South African guidelines (March 2012–April 2013: CD4≤350 cells/µL or WHO stage IV; April 2013–January 2015: CD4≤350 cells/µL or WHO stage III/IV or pregnant women or tuberculosis co-infected). TasP trial participants can also access HIV and ART care in the Department of Health (DoH clinics).

The TasP trial started in 10 (2×5) clusters from March 2012 to July 2014, with a further 12 (2×6) clusters from July 2014, bringing the total number of clusters to 22 (2×11) at full implementation. Each cluster is composed of an average of about 1000 residents ≥16 years. Data from the first 10 clusters were used for this analysis.

### Study population

We included all residents aged ≥16 years from both arms of the trial who were (i) contacted by a fieldworker, (ii) ascertained HIV positive (positive rapid HIV test result or self-reported to be HIV positive), (iii) referred to a TasP clinic between March 2012 and June 2014, and (iv) never been or not in HIV care at the time of referral (i.e. no CD4 count or viral load measurements in the DoH or TasP clinics in the 13 months before referral).

We excluded individuals with inconsistent dates (date of first clinic visit, death or out-migration before the date of first referral), as well as those with a period of observation shorter than three months. We focused statistical analyses on individuals without missing data for explanatory variables.

### Outcome and explanatory variables

The outcome was linkage to HIV care following HIV diagnosis within three months of first referral in individuals who had never or not recently been in HIV care. Linkage to care was defined as attending a TasP clinic (the variable used was date of visit) or a DoH clinic (the variable used was date of last CD4 count or viral load measurement), after matching between the TasP trial database and the ARTemis database. We obtained ethics approval to match the TasP trial database with the DoH HIV care and treatment database (ARTemis), both developed and hosted at the Africa Centre [23]. Matching was based on South African ID number, first names, last names, dates of birth and cell phone numbers. The period of three months (i.e. 91 days) between first referral and linkage to care was chosen in accordance with Fox *et al*. [26].

Matching between TasP and ARTemis databases was also used to define the variable “HIV care status at referral” with four categories: (i) newly diagnosed (positive rapid HIV test through HBHCT, no self-report of HIV diagnosis and not in the ARTemis database before referral); (ii) already diagnosed but never accessed HIV care in the local HIV programme (self-reported HIV positive through HBHCT, not in the ARTemis database before referral); (iii) already accessed HIV care in the local HIV programme but considered lost-to-follow-up (LTFU) for 13 to 24 months (in ARTemis database before first referral but no CD4 count or viral load measurements in the DoH or TasP clinics in the 13–24 months before referral); or (iv) LTFU for more than 24 months.

Further explanatory socio-demographic and HIV-related variables were based on questionnaires administered face to face by trained interviewers during the repeat home-based visits; we considered information from the home visit before and closest to the date of first referral. We also included trial calendar round (CR) of HBHCT at referral (CR1: identification of HIV infection at the first home visit by HIV fieldworkers; CR2/CR3: identification of HIV infection at the second or the third home visit [individuals identified HIV positive in CR2/CR3 could be those not tested for HIV during the first round (CR1) because they were not at home, they refused to be tested, they seroconverted between rounds or they had just become eligible because they turned 16 years old]).

### Statistical analysis

Linkage to HIV care following HIV diagnosis was described with Kaplan–Meier curves stratified by sex. The association between sex and linkage to HIV care was estimated using a log-rank test. Univariable and multivariable logistic regression models were conducted to explore factors associated with linkage to HIV care within three months of referral. For ordinal variables, a test for trend was also conducted. Multivariable analysis was adjusted for sex and trial arm, and included variables associated with linkage to HIV care with a *p*-value <0.20 in univariable analysis. The interactions with sex and HIV care status at referral were tested, but no interactions were found (Supplementary Table 1). Analyses were carried out using STATA version 13.0 (StataCorp, College Station, Texas).

### Ethical approval

The trial was approved by the Biomedical Research Ethics Committee (BREC) of the University of KwaZulu-Natal (BFC 104/11) and the Medicines Control Council of South Africa.

Our consent procedures include at home level, for each survey round, verbal consent of the homestead's owner and of the head of household, as well as written individual consent. For participants aged 16 or 17, we collect both the consent of the participant and the consent of a parent or a guardian.

## Results

**Figure 1:**
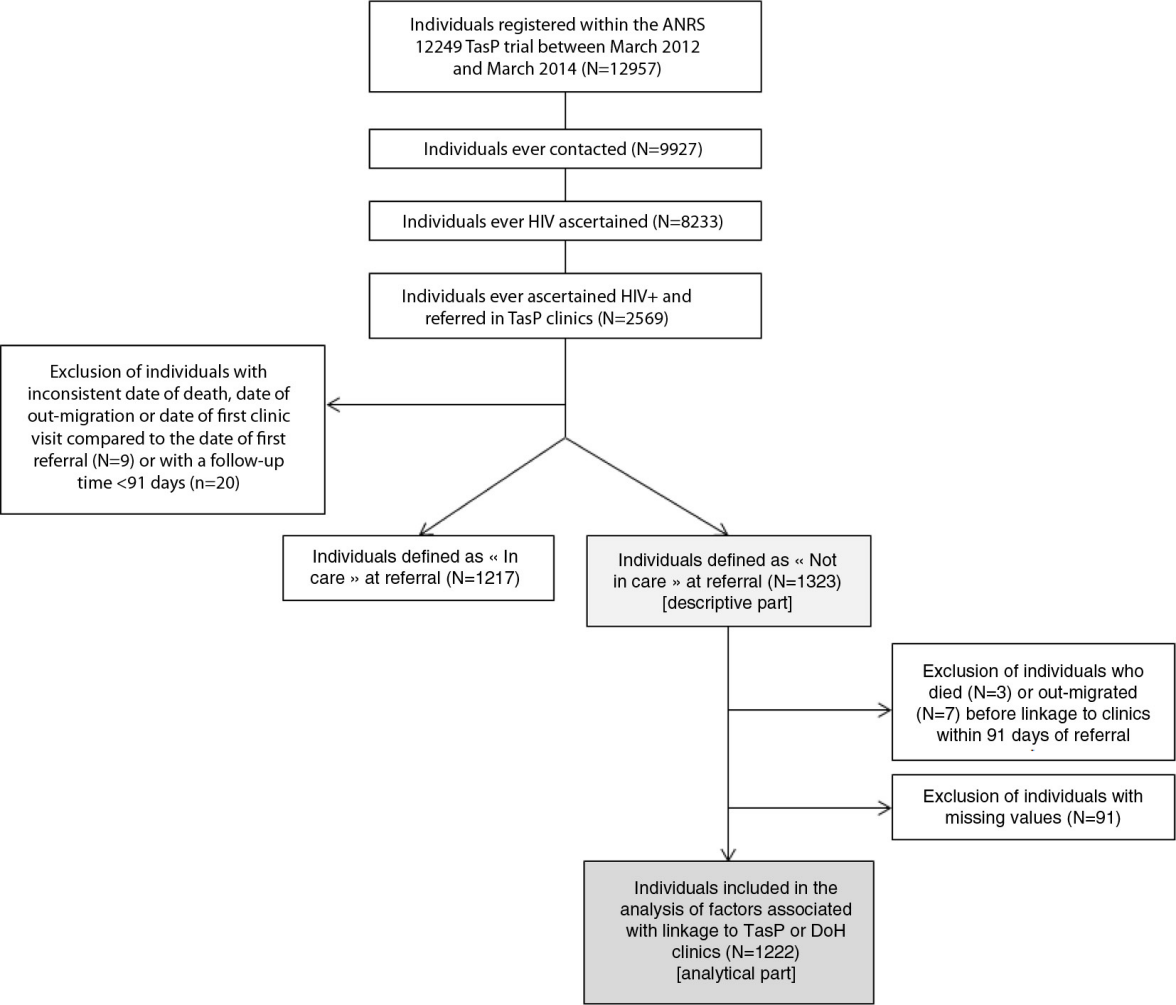
Flowchart of the cohort, ANRS TasP trial, rural South Africa, 2012–2014.

Of the 12,957 adults registered in the TasP trial, 9927 were ever contacted, of whom 8233 had their HIV status ascertained. Of these, 2569 (31.2%) were identified HIV positive and referred to a TasP clinic (Figure 1); and among them, 29 adults were excluded because they had inconsistent dates (*n*=9) or their period of observation was <3 months (*n*=20). Of the remaining 2540 adults, 1323 were considered “never or not currently in care,” of whom 72.9% (*n*=965) were women.

At the time of referral, about 43% of included adults were newly diagnosed and 26% had previously been diagnosed but had never accessed care (Table 1); about 31% of adults had already accessed HIV care in the local HIV programme but were considered LTFU, half of them for >24 months. About 60% of HIV-positive adults were identified as HIV positive in the first round of fieldwork (Table 1, trial characteristics).

**Table 1:** Description of the study population at referral, ANRS TasP trial, rural South Africa, 2012–2014 (*N*=1323)

	Total (*N*=1323)		Women (*N*=965)		Men (*N*=358)	
			
	*n*	(%)	*n*	(%)	*n*	(%)
HIV care status at referral						
Never in care, newly diagnosed	567	(42.9)	381	(39.5)	186	(52.0)
Never in care, already diagnosed	346	(26.1)	247	(27.6)	79	(22.1)
LTFU >24 months	202	(15.3)	161	(16.7)	41	(11.4)
LTFU 13–24 months	208	(15.7)	156	(16.2)	52	(14.5)
Socio-demographic characteristics						
Age at referral (years)						
16–19	78	(5.9)	69	(7.1)	9	(2.5)
20–29	464	(35.1)	356	(36.9)	108	(30.2)
30–39	355	(26.8)	242	(25.1)	113	(31.6)
40–49	189	(14.3)	127	(13.2)	62	(17.3)
50–84	193	(14.6)	139	(14.4)	54	(15.1)
Missing	44	(3.3)	32	(3.3)	12	(3.3)
Education level						
Primary or less	491	(37.1)	342	(35.4)	149	(41.6)
Some secondary	440	(33.3)	320	(33.2)	120	(33.5)
At least completed secondary	383	(28.9)	297	(30.8)	86	(24.0)
Missing	9	(0.7)	6	(0.6)	3	(0.9)
Occupational status						
Employed	210	(15.9)	120	(12.4)	90	(25.1)
Student	109	(8.2)	89	(9.2)	20	(5.6)
Not student, not employed	985	(74.5)	741	(76.8)	244	(68.2)
Missing	19	(1.4)	15	(1.6)	4	(1.1)
Household wealth assets^a^						
Low	471	(35.6)	350	(36.3)	121	(33.8)
Middle	554	(41.9)	408	(42.3)	146	(40.8)
High	287	(21.7)	198	(20.5)	89	(24.9)
Missing	11	(0.8)	9	(0.9)	2	(0.5)
Characteristics relating to HIV knowledge and perception						
Knowing HIV-positive family member						
No	821	(62.5)	565	(59.2)	256	(71.5)
Yes	491	(37.1)	391	(40.5)	100	(27.9)
Missing	5	(0.4)	3	(0.3)	2	(0.6)
Would take ARV if diagnosed HIV positive						
Agree	1216	(91.9)	876	(90.8)	340	(95.0)
Disagree	63	(4.8)	56	(5.8)	7	(2.0)
Don't know	25	(1.9)	18	(1.9)	7	(2.0)
Missing	19	(1.4)	15	(1.5)	4	(1.0)
Think that people avoid HIV-positive individuals						
Agree	470	(35.5)	356	(36.9)	114	(31.8)
Disagree	697	(52.7)	496	(51.4)	201	(56.2)
Don't know	137	(10.4)	100	(10.4)	37	(10.3)
Missing	19	(1.4)	13	(1.3)	6	(1.7)
Think that people don't blame HIV-positive individuals						
Agree	707	(53.4)	515	(53.4)	192	(53.6)
Disagree	448	(33.9)	330	(34.2)	118	(33.0)
Don't know	153	(11.6)	110	(11.4)	43	(12.0)
Missing	15	(1.1)	10	(1.0)	5	(1.4)
Trial characteristics						
Distance from home to the closest TasP clinic						
<1 km	486	(36.7)	355	(36.8)	131	(36.6)
1–2 km	468	(35.4)	343	(35.5)	125	(34.9)
2–5 km	369	(27.9)	267	(27.7)	102	(28.5)
Calendar round at referral						
CR1	793	(59.9)	590	(61.1)	203	(56.7)
CR2/CR3	530	(40.1)	375	(38.9)	155	(43.3)
Trial arm						
Control	717	(54.2)	535	(55.4)	182	(50.8)
Intervention	606	(45.8)	430	(44.6)	176	(49.2)

LTFU, lost-to-follow-up; ARV, antiretroviral; CR, calendar round at referral.

a Household wealth assets had been defined in three categories (low, middle and high) in agreement with a principal component analysis considering sources of energy, amenities and access to drinking water and toilet facilities in this populations [27].

The included population was relatively young (44% of women and 32% of men were <30 years) and with a low education level (35% of women and 41% of men did not go to secondary school). A large proportion of the population was neither employed nor studying (>76% of women and >68% of men). Almost 41% of women and 28% of men declared that they knew at least one other family member who was HIV positive. About one-third of the included population perceived stigma against HIV-positive individuals (>35% agreed that people of the community avoid HIV-positive individuals and almost 34% disagreed that people of the community don't blame HIV-positive individuals). More than 90% of men and women reported they would take ARVs “as soon as possible” if diagnosed HIV positive (Table 1).

### Linkage to care proportion

Overall, 36.9% of included adults never or not currently in HIV care at the time of referral were linked to care (in either TasP or DoH clinic) within three months of HBHCT (Table 2).

**Table 2:** Linkage to HIV care within three months of referral, ANRS TasP trial, rural South Africa, 2012–2014 (*N*=1323)

	Total (*N*=1323)		Women (*N*=965)		Men (*N*=358)	
			
	*n*	%	*n*	%	*n*	%
Linkage to clinics	488	36.9	349	36.2	139	38.8
*Linkage to TasP clinic only*	*381*	*28.8*	*267*	*27.7*	*114*	*31.8*
*Linkage to DoH then to TasP clinics*	*39*	*3.0*	*28*	*2.9*	*11*	*3.1*
*Linkage to TasP then to DoH clinics*	*7*	*0.5*	*4*	*0.4*	*3*	*0.8*
*Linkage to DoH clinic only*	*61*	*4.6*	*50*	*5.2*	*11*	*3.1*
Death	3	0.2	3	0.3	0	0.0
Out-migration	7	0.5	7	0.7	0	0.0
No linkage to clinics	825	62.4	606	62.8	219	61.2

DoH, Department of Health; TasP, treatment as prevention.

**Figure 2:**
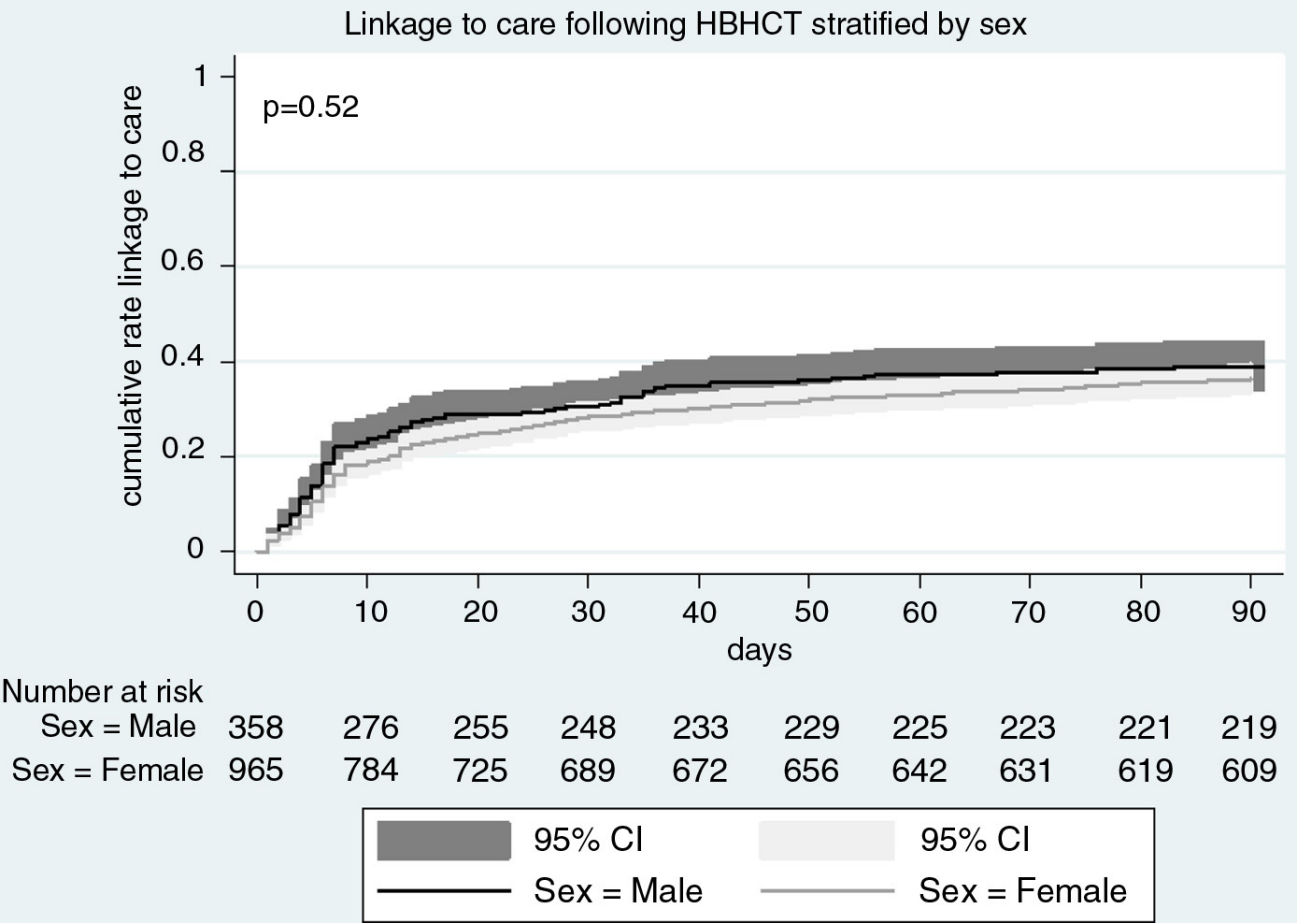
Cumulative incidence of linkage to TasP or DoH clinics within three months of referral, stratified by sex, ANRS TasP trial, rural South Africa, 2012–2014 (*N*=1323). DoH: Department of Health; TasP: treatment as prevention.

Linkage to HIV care occurred mostly during the first month after referral then increased slowly over time, with no significant differences by sex (Figure 2).

### Factors associated with linkage to HIV care within three months of referral

For this analysis, we excluded individuals who died (*n*=3) or had out-migrated (*n*=7) within three months of referral, as well as those with missing values (*n*=91). In total, 1222 individuals were thus included (Figure 1); included individuals were more likely to have had previous contact with the local HIV programme than those excluded before HIV identification within the TasP trial (20.8% vs. 31.9%, *p*=0.002, Supplementary Table 2).

HIV care status at referral was significantly associated with linkage to HIV care (Table 3): 32.1% of individuals newly diagnosed were linked to HIV care within three months of referral, compared with 42.8% among those who had already accessed HIV care previously but were LTFU for >24 months at the time of referral linked to HIV care (adjusted odd ratio [aOR]=1.44, 95% confidence interval [CI] =1.00–2.06), and 56.9% among those LTFU for more than 13 to 24 months (aOR=0.2.52, CI=1.77–3.61). No significant difference in linkage to care percentages was observed between individuals who were newly diagnosed and those already diagnosed but having never been in care.

**Table 3:** Factors associated with linkage to TasP or DoH clinics within three months of referral, ANRS TasP trial, rural South Africa, 2012–2014 (*N*=1222 – complete data)

			Univariable analysis			Multivariable analysis^a^		
				
	*n*	% linkage	OR	95%CI	p*	aOR	95%CI	p*
HIV care status at referral								
Never in care, newly diagnosed	529	32.1	1.00	–	<0.001	1.00	–	<0.001
Never in care, already diagnosed	304	31.6	0.97	0.72–1.32		0.97	0.71–1.34	
LTFU >24 months	194	42.8	1.58	1.13–2.21		1.44	1.00–2.06	
LTFU 13–24 months	195	56.9	2.79	1.99–3.90		2.52	1.77–3.61	
Socio-demographic characteristics								
Sex								
Men	336	39.6	1.00	–	0.39	1.00	–	0.47
Women	886	36.9	0.89	0.69–1.16		0.90	0.68–1.20	
Age at referral (years)								
16–19	75	20.0	0.54	0.30–0.99	<0.001	0.77	0.40–1.48	0.40
20–29	437	31.6	1.00	–		1.00	–	
30–39	340	38.2	1.34	1.00–1.81		1.09	0.79–1.50	
40–49	185	44.3	1.72	1.21–2.46		1.16	0.77–1.74	
50–84	185	51.4	2.29	1.61–3.25		1.47	0.94–2.28	
Education level								
Primary or less	455	47.7	1.00	–	<0.001	1.00	–	0.01
Some secondary	407	33.7	0.56	0.42–0.73		0.68	0.49–0.96	
At least completed secondary	360	29.4	0.46	0.34–0.61		0.59	0.41–0.84	
Occupational status								
Employed	200	42.5	1.00	–	<0.001	1.00	–	0.07
Student	102	16.7	0.27	0.15–0.49		0.47	0.24–0.92	
Not student, not employed	920	38.9	0.86	0.63–1.18		0.94	0.67–1.31	
Household wealth assets								
Low	439	37.1	1.00	–	0.62			
Middle	514	39.1	1.09	0.84–1.41				
High	269	35.7	0.94	0.69–1.29				
Characteristics relating to HIV knowledge and perception								
Knowing HIV-positive family member								
No	764	35.0	1.00	–	0.01	1.00	–	0.004
Yes	458	42.1	1.36	1.07–1.72		1.45	1.12–1.86	
Would take ARV is diagnosed HIV positive								
Agree	1141	38.5	1.83	1.01–3.34	0.06	2.16	1.13–4.10	0.03
Disagree	59	25.4	1.00	–		1.00	–	
Don't know	22	27.3	1.10	0.36–3.33		1.18	0.37–3.76	
Think that people avoid HIV-positive individuals								
Agree	446	36.8	0.91	0.71–1.16	0.39			
Disagree	652	39.1	1.00	–				
Don't know	124	33.1	0.77	0.51–1.15				
Think that people don't blame HIV-positive individuals								
Agree	661	38.1	1.03	0.80–1.32	0.87			
Disagree	424	37.5	1.00	–				
Don't know	137	35.8	0.93	0.62–1.39				
Trial characteristics								
Distance to the closest TasP clinic								
0–1 km	447	45.0	1.00	–	<0.001	1.00	–	<0.001
1–2 km	433	33.7	0.62	0.47–0.82		0.58	0.44–0.78	
2–5 km	342	33.0	0.60	0.45–0.81		0.57	0.41–0.77	
Calendar round at referral								
CR1	734	41.3	1.00	–	0.001	1.00	–	0.03
CR2/CR3	488	32.2	0.67	0.53–0.86		0.75	0.58–0.97	

OR, odd ratio; 95%CI, 95% confidence interval; aOR, adjusted odd ratio; LTFU, lost-to-follow-up; ARV, antiretroviral; CR, calendar round at referral; DoH, Department of Health; TasP, treatment as prevention.

* *p*, likelihood ratio test *p*-values.

a Variables included in the multivariable model: HIV care status, sex, age, education level, occupational status, knowing HIV-positive family member, ARV if diagnosed HIV positive, distance to clinic, trial calendar round.

Linkage to care significantly decreased with education level (*p* for trend<0.001) and was associated with occupational status: 16.7% of students linked to HIV care compared to 42.5% of employed (aOR=0.47, CI=0.24–0.92). However, linkage to care did not differ significantly between employed individuals and those who were neither student nor employed.

Further, percentages of linkage to care were higher in individuals who declared knowing at least another HIV-positive family member (42.1% vs. 35.0% among those who did not know another HIV-positive family member, aOR=1.45, CI=1.12–1.86), as well as in those who stated they would agree taking ARVs “as soon as possible” if diagnosed HIV positive (38.5% vs. 25.4% among those who didn't agree, aOR=2.16, CI=1.13–4.10).

Living closer to a TasP clinic was significantly associated with increased linkage to care (*p* for trend <0.001). Finally, adults who were ascertained HIV positive and referred to a TasP clinic in the second or the third round of trial fieldwork were less likely to be linked to HIV care (32.2% vs. 41.3%, aOR=0.75, CI=0.58–0.97) (Table 3).

The percentages of linkage to care also increased significantly with age (*p* trend <0.001), but this association lost significance in the multivariable model. Household wealth and stigma-related variables were not significantly associated with linkage to care.

## Discussion

In this rural area, fewer than 40% of individuals identified HIV positive through HBHCT in the TasP trial, and who had never or were not currently in HIV care, accessed an HIV clinic within three months of referral. These results are in line with a previous study in Kenya (42% linkage to care following HBHCT) [28] but lower than seen in other studies in rural South Africa (62%) [19], Uganda and South Africa (86%) [29]. However, it is difficult to compare these studies as definitions of linkage to care varied (especially regarding the time between HIV diagnosis and linkage to care used in these studies) [19]. We also observed that linkage to care, if it happened, was most likely in the first month after referral; we hypothesize that this pattern, also seen elsewhere [19], is suggestive of an element of being ready to engage with HIV care and treatment, and where this fits in terms of personal priorities.

Linkage to HIV care did not significantly differ according to sex, a finding consistent with the Kenya study [28]. We identified several other factors associated with linkage to HIV care. The first was HIV care status at referral, which allows for previous HIV diagnosis and care. Adults who had never been in HIV care when identified as HIV positive during the TasP trial were significantly less likely to link to HIV care than those who had previously been in HIV care but had been LTFU. This may suggest that the latter may have already come to terms with their HIV diagnosis and may possibly already have disclosed their HIV status to relatives and friends; it has indeed previously been reported that HIV disclosure is associated with access to HIV care following HBHCT in Kenya [28]. Further, individuals who were previously in care may have a better understanding of what is involved in HIV care than those who never accessed HIV care and may thus be more inclined to re-engage with HIV care when provided with a convenient alternative which is closer to them as in the TasP trial. Among people who had never been in care, there were no differences in linkage to care whether people were newly diagnosed in the trial or not, which was also found in Kenya [28]; these findings suggest that, beyond the need for time required to process an HIV-positive status following diagnosis [30,31], there are additional challenges for linkage to care which should be explored further.

While stigma variables, as collected within the trial, were not significantly associated with linkage to HIV care, people who knew another family member to be HIV positive were more likely to access HIV care than those who did not. This could suggest that people with HIV history in the family may have a better knowledge of HIV care and treatment and may thus be more disposed to access HIV care themselves when they are referred. They may also be more confident in disclosing their HIV status, and trust they will receive family support. This is especially important in a context where HIV care is offered in dedicated services or clinics, separated from general care, where patients are easily identified as HIV positive, as it is the case in this TasP trial as well as in the local HIV programme.

Linkage to care was also especially low in students (16.7%); these individuals may be less economically and logistically independent and may not consider HIV care a priority.

We also showed that people who were identified HIV positive at the first home-based contact were significantly more likely to link to HIV care than those identified HIV positive only at second or third contact. People referred to care for the first time only after several contacts may either be recent HIV seroconverters, needing time to accept their HIV status, or who refused HIV testing initially, and who, we hypothesize, may be less inclined to access care once diagnosed than those readily accepting an HIV test at the first contact.

Linkage to HIV care was lower in people with higher education levels. A possible suggestion for this may include being able to afford HIV care outside of the trial if closer to their place of employment (private sector, clinics outside of the areas). Higher education level may also be associated with being at work or engaged in other productive activity, which could have hindered clinic attendance.

Linkage to HIV care also decreased significantly with distance to clinic. Although everyone lived within a 45-minute walking distance to the trial clinic in their cluster, those who were even closer to the clinic, and required less travel time to access it, were more likely to link to care than those who lived further away. We hypothesize that the role of distance to clinic in linkage to care would be even more important in settings where HIV clinics are not as accessible as in the TasP trial area. Economic and logistic barriers associated with distance to clinic would need further investigation.

Finally, while it has not been considered in this study, an association between perception of health status and linkage to HIV care following HBHCT has been observed in other settings in Kenya [28] and South Africa [19]. For a long time in these countries, the public health message has been to wait for the CD4 count to drop below a certain threshold before being able to initiate ART. With the 2015 WHO recommendation of ART initiation to all HIV-positive individuals regardless of clinical or immunological status, public health messaging will need to change with specific education and counselling at community and individual levels.

Given the number of individual-level factors associated with a link to HIV care in our study, strategies of patient-centred HIV care with specific social support should be considered [32]. The engagement of health system navigators who can call, visit people at home or in neutral places and, if needed, escort them to clinic could also be proposed [33,34] although this was not found to be successful in urban South Africa [35,36]. For people who face difficulties in accessing a clinic, home-based ART initiation with home-based care could be implemented [37] although it remains important to evaluate the impact of such an intervention on long-term follow-up.

Our study has some methodological limitations. First, it was nested in a large randomized trial with specific trial clinics providing higher coverage of HIV care than would ordinarily be available within a standard HIV programme. Another limitation is that we do not know whether people linked to care outside of both the TasP trial and the local HIV programme, which could have led to an underestimation of the proportion of linkage to HIV care. However, such an underestimation is unlikely to be substantial as our definition of linkage to care considers a short time period (three months) following referral during which people from this relatively poor area would have found difficult to access HIV care outside the area. Finally, we excluded about 10% of the sample (who had missing data) in the analysis of factors associated with linkage to HIV care; included individuals may not be representative of the overall population as they were more likely to have previously sought HIV care in the local HIV programme before TasP trial implementation, than excluded people. However, we did not observe other statistically significant differences between included and excluded participants, and the population size was sufficient to statistically test factors associated with linkage to HIV care in the trial.

## Conclusions

A major strength is that the study has been conducted prospectively within a large population. The results are particularly relevant in the context of rapidly evolving HIV care. Indeed, in the light of the recent findings from the TEMPRANO and START trials [1,2] demonstrating the strong clinical benefits of early ART at an individual level, international HIV guidelines have been expanded towards a universal test and treat strategy [8]. The critical remaining questions relate to how best to ensure operational implementation of such a strategy at a population level in order to achieve the UNAIDS 90–90–90 target [9]. Our results show that an HBHCT intervention is useful not only to diagnose HIV-positive individuals who do not know their HIV status, but also to re-engage people previously in care but LTFU. We also highlighted the challenges inherent in achieving the second “90%” of the UNAIDS target: the number of people linked to HIV care and initiating ART must be substantially increased with combined interventions at the patient-level. Such strategies will need to be fully evaluated in different settings, including ours, at population level.

## Supplementary Material

Access to HIV care in the context of universal test and treat: challenges within the ANRS 12249 TasP cluster-randomized trial in rural South Africa

## Appendix

**Table:** Composition of the TasP Study Group

Name	Role	Affiliation
**Investigators**		
François Dabis	Co-PI (France)	Univ. Bordeaux, ISPED, Centre Inserm U897 – Epidemiologie-Biostatistique, Bordeaux, France
		INSERM, ISPED, Centre Inserm U897 – Epidemiologie-Biostatistique, Bordeaux, France
Marie-Louise Newell	Co-PI (United Kingdom)	Africa Centre for Health and Population Studies, University of KwaZulu-Natal, South Africa Faculty of Medicine, University of Southampton, UK
Deenan Pillay	Co-PI (South Africa)	Africa Centre for Health and Population Studies, University of KwaZulu-Natal, South Africa Faculty of Medical Sciences, University College London, UK
**Coordinators**		
Collins Iwuji	Trial Coordinator and HIV Physician (South Africa)	Africa Centre for Health and Population Studies, University of KwaZulu-Natal, South Africa Research Department of Infection and Population Health, University College London, UK
Joanna Orne-Gliemann	Trial Coordinator (France)	Univ. Bordeaux, ISPED, Centre Inserm U897 – Epidemiologie-Biostatistique, Bordeaux, France INSERM, ISPED, Centre Inserm U897 – Epidemiologie-Biostatistique, Bordeaux, France
**Study team**		
Till Bärnighausen	Health economics	Africa Centre for Health and Population Studies, University of KwaZulu-Natal, South Africa
		Dept of Global Health & Population, Harvard School of Public Health, Harvard Univ. Boston
Eric Balestre	Epidemiology and Biostatistics	Univ. Bordeaux, ISPED, Centre Inserm U897 – Epidemiologie-Biostatistique, Bordeaux, France INSERM, ISPED, Centre Inserm U897 – Epidemiologie-Biostatistique, Bordeaux, France
Sylvie Boyer	Health economics	INSERM, UMR912 (SESSTIM), Marseille, France
		Aix Marseille Université, UMR_S912, IRD, Marseille, France
		ORS PACA, Observatoire Régional de la Santé Provence-Alpes-Côte d'Azur, Marseille, France
Alexandra Calmy	Adult Medicine	Service des maladies infectieuses, Hôpital Universitaire de Geneve, Genève.
Vincent Calvez	Virology	Department of virology, Hôpital Pitié-Salpétrière, Paris, France
Marie-Laure Chaix	Virology	EA 3620, Université Paris-Descartes, Laboratoire de Virologie, Hôpital Necker-Enfants Malades, AP-HP, Paris
Rosemary Dray-Spira	Social sciences	INSERM U1018, CESP, Epidemiology of Occupational and Social Determinants of Health, Villejuif, France
		University of Versailles Saint-Quentin, UMRS 1018, Villejuif, France
Kamal ElFarouki	Social sciences	INSERM U1018, CESP, Epidemiology of Occupational and Social Determinants of Health, Villejuif, France
		University of Versailles Saint-Quentin, UMRS 1018, Villejuif, France
Kenneth Freedberg	Modelling	Massachusetts General Hospital, Harvard Medical School, Boston, MA, USA.
Kobus Herbst	Data management	Africa Centre for Health and Population Studies, University of KwaZulu-Natal, South Africa
John Imrie	Social sciences	Futures Group, Johannesburg, South Africa
		Centre for Sexual Health and HIV Research, Research Department of Infection and Population, Faculty of Population Health Sciences, University College London, London, UK
Sophie Karcher	Data management	Univ. Bordeaux, ISPED, Centre Inserm U897 – Epidemiologie-Biostatistique, Bordeaux, France
		INSERM, ISPED, Centre Inserm U897 – Epidemiologie-Biostatistique, Bordeaux, France
Joseph Larmarange	Social sciences	CEPED (Centre Population & Développement-UMR 196-Paris Descartes/INED/IRD), IRD (Institut de Recherche pour le Développement), Paris, France.
		Africa Centre for Health and Population Studies, University of KwaZulu-Natal, South Africa
France Lert	Social Sciences	INSERM U1018, CESP, Epidemiology of Occupational and Social Determinants of Health, Villejuif, France
		University of Versailles Saint-Quentin, UMRS 1018, Villejuif, France
Richard Lessells	Adult medicine	London School of Hygiene and Tropical Medicine, UK
Thembisa Makowa	Field operations	Africa Centre for Health and Population Studies, University of KwaZulu-Natal, South Africa
Anne-Geneviève Marcelin	Virology	Department of virology, Hôpital Pitié-Salpétrière, Paris, France
Laura March	Health economics	INSERM, UMR912 (SESSTIM), Marseille, France
		Aix Marseille Université, UMR_S912, IRD, Marseille, France
		ORS PACA, Observatoire Régional de la Santé Provence-Alpes-Côte d'Azur, Marseille, France
Nuala McGrath	Epidemiology/Social sciences	Academic Unit of Primary Care and Population Sciences, and Department of Social statistics and Demography, University of Southampton, United Kingdom
Kevi Naidu	Adult medicine	Africa Centre for Health and Population Studies, University of KwaZulu-Natal, South Africa
Colin Newell	Data management	Africa Centre for Health and Population Studies, University of KwaZulu-Natal, South Africa
Nonhlanhla Okesola	Nurse manager	Africa Centre for Health and Population Studies, University of KwaZulu-Natal, South Africa
Tulio de Oliveira	Bioinformatics	Africa Centre for Health and Population Studies, University of KwaZulu-Natal, South Africa
Melanie Plazy	Epidemiology/social sciences	Univ. Bordeaux, ISPED, Centre Inserm U897- Epidemiologie-Biostatistique, Bordeaux, France
		INSERM, ISPED, Centre Inserm U897- Epidemiologie-Biostatistique, Bordeaux, France
Tamsen Rochat	Anthropology/psychology	Africa Centre for Health and Population Studies, University of KwaZulu-Natal, South Africa
Bruno Spire	Health economics	INSERM, UMR912 (SESSTIM), 13006, Marseille, France
		Aix Marseille Université, UMR_S912, IRD, Marseille, France
		ORS PACA, Observatoire Régional de la Santé Provence-Alpes-Côte d'Azur, Marseille, France
Frank Tanser	Epidemiology and Biostatistics	Africa Centre for Health and Population Studies, University of KwaZulu-Natal, South Africa
Rodolphe Thiébaut	Epidemiology and Biostatistics	Univ. Bordeaux, ISPED, Centre Inserm U897 – Epidemiologie-Biostatistique, Bordeaux, France INSERM, ISPED, Centre Inserm U897 – Epidemiologie-Biostatistique, Bordeaux, France
Johannes Viljoen	Virology	Africa Centre for Health and Population Studies, University of KwaZulu-Natal, South Africa
Thembelile Zuma	Psychology/Social sciences	Africa Centre for Health and Population Studies, University of KwaZulu-Natal, South Africa

**Scientific advisory board**

Chair: Bernard Hirschel (Switzerland)

International experts: Xavier Anglaret (Ivory Coast), Hoosen Cooavdia (South Africa), Alpha Diallo (France), Bruno Giraudeau (France), Jean-Michel Molina (France), Lynn Morris (South Africa), François Venter (South Africa), Sibongile Zungu (South Africa)

Community representatives: Eric Fleutelot (France), Eric Goemaere (South Africa), Calice Talom (Cameroon)

Sponsor representatives (ANRS): Brigitte Bazin, Claire Rekacewicz

Pharmaceutical company representatives: Golriz Pahlavan-Grumel (MSD), Alice Jacob (Gilead)

**Data safety and monitoring board**

Chair: Patrick Yeni (France)

Members: Sinead Delany-Moretlwe (South Africa), Nathan Ford (South Africa), Catherine Hankins (Netherlands), Helen Weiss (UK)
