# Supplementary material for: Access to HIV care in the context of universal test and treat: challenges within the ANRS 12249 TasP cluster-randomized trial in rural South Africa
Source: J Int AIDS Soc. 2016 Jun 1;19(1):20913. doi: 10.7448/IAS.19.1.20913 (PMC4891946; doi:10.7448/IAS.19.1.20913)
Supplement: Access to HIV care in the context of universal test and treat: challenges within the ANRS 12249 TasP cluster-randomized trial in rural South Africa [file JIAS-19-20913-s001.pdf]

APPENDIX 1.

Table S1. P-value of interaction with sex and with HIV care status at referral (N=1218 – complete data)

Variable	p-value interaction Sex	p-value interaction Status at referral
HIV care status at referral	0.81	/
Sex	/	0.81
Age at referral	0.11	0.05
Education level	0.64	0.12
Occupational status	0.67	0.73
Household wealth assets	0.17	0.17
Knowing HIV+ family member	0.89	0.88
Would take ARV if diagnosed HIV+	0.99	0.79
Think that people avoid HIV+ individuals	0.80	0.78
Think that people don’t blame HIV+ individuals	0.99	0.12
Distance to the closest TasP clinic	0.44	0.32
Calendar round at referral	0.56	0.36

APPENDIX 2.

Table S2. Baseline variables associated with inclusion/exclusion status

	**Included (N=1222)**		**Excluded**		**p**
	**n**	**(%)**	**n**	**(%)**	
**HIV care status at referral**			*(N=101)*		
Never in care, newly diagnosed	529	(42.0)	38	(37.6)	*0.002*
Never in care, already diagnosed	304	(25.0)	42	(41.6)	
LTFU >24 months	194	(15.9)	8	(7.9)	
LTFU 13-24 months	195	(16.0)	13	(12.9)	
**Sex**			*(N=101)*		
Male	336	(27.5)	22	(21.8)	*0.21*
Female	886	(72.5)	79	(78.2)	
**Age at referral (years)**			*(N=57)*		
16-19	75	(6.1)	3	(5.3)	*0.33*
20-29	437	(35.8)	27	(47.4)	
30-39	340	(27.9)	15	(26.3)	
40-49	185	(15.3)	4	(7.0)	
50-84	185	(15.1)	8	(14.0)	
**Education level**			*(N=92)*		
Primary or less	455	(37.2)	36	(39.1)	*0.66*
Some secondary	407	(33.3)	33	(35.9)	
At least completed secondary	360	(29.5)	23	(25.0)	
**Occupational status**			*(N=82)*		
Employed	200	(16.4)	10	(12.2)	*0.61*
Student	102	(8.3)	7	(8.5)	
Inactive	920	(75.3)	65	(79.3)	
**Household wealth assets**			*(N=90)*		
Low	438	(35.8)	31	(34.4)	*0.91*
Middle	515	(42.2)	40	(44.5)	
High	269	(22.0)	19	(21.1)	
**Knowing HIV+ family member**			*(N=96)*		

No	764	(62.5)	63	(65.6)	*0.55*
Yes	458	(37.5)	33	(34.4)	
**Would take ARV if diagnosed HIV+**			*(N=82)*		
Agree	1141	(93.4)	75	(91.5)	*0.49*
Disagree	59	(4.8)	4	(4.9)	
Don't know	22	(1.8)	3	(3.7)	
**Think that people avoid HIV+ individuals**			*(N=82)*		
Agree	446	(36.5)	24	(29.3)	*0.17*
Disagree	652	(53.4)	45	(54.9)	
Don't know	124	(10.1)	13	(15.8)	
**Think that people don't blame HIV+ individuals**			*(N=86)*		
Agree	661	(54.1)	46	(53.5)	*0.09*
Disagree	424	(34.7)	24	(27.9)	
Don't know	137	(11.2)	16	(18.6)	
**Distance from home to the closest TasP clinic**			*(N=101)*		
0-1 km	447	(36.6)	39	(38.6)	*0.92*
1-2 km	433	(35.4)	35	(34.7)	
2-5 km	342	(28.0)	27	(26.7)	
**Calendar Round at referral**			*(N=101)*		
CR1	734	(60.1)	59	(58.4)	*0.75*
CR2/CR3	488	(39.9)	42	(41.6)	
**Trial arm**			*(N=101)*		
Control	672	(55.0)	45	(44.5)	*0.05*
Intervention	550	(45.0)	56	(55.5)	

APPENDIX 3.

Table S3. Composition of the TasP Study Group

Name	Role	Affiliation
**Investigators**		
François Dabis	Co-PI (France)	- Univ. Bordeaux, ISPED, Centre Inserm U897- Epidemiologie-Biostatistique, Bordeaux, France<br>- INSERM, ISPED, Centre Inserm U897- Epidemiologie-Biostatistique, Bordeaux, France
Marie-Louise Newell	Co-PI (United Kingdom)	- Africa Centre for Health and Population Studies, University of KwaZulu-Natal, South Africa<br>- Faculty of Medicine, University of Southampton, UK
Deenan Pillay	Co-PI (South Africa)	- Africa Centre for Health and Population Studies, University of KwaZulu-Natal, South Africa<br>- Faculty of Medical Sciences, University College London, UK
**Coordinators**		
Collins Iwuji	Trial Coordinator and HIV Physician (South Africa)	- Africa Centre for Health and Population Studies, University of KwaZulu-Natal, South Africa<br>- Research Department of Infection and Population Health, University College London, UK
Joanna Orne-Gliemann	Trial Coordinator (France)	- Univ. Bordeaux, ISPED, Centre Inserm U897- Epidemiologie-Biostatistique, Bordeaux, France<br>- INSERM, ISPED, Centre Inserm U897- Epidemiologie-Biostatistique, Bordeaux, France
**Study team**		
Till Bärnighausen	Health economics	- Africa Centre for Health and Population Studies, University of KwaZulu-Natal, South Africa<br>- Dept of Global Health & Population, Harvard School of Public Health, Harvard Univ. Boston
Eric Balestre	Epidemiology and Biostatistics	- Univ. Bordeaux, ISPED, Centre Inserm U897- Epidemiologie-Biostatistique, Bordeaux, France<br>- INSERM, ISPED, Centre Inserm U897- Epidemiologie-Biostatistique, Bordeaux, France
Sylvie Boyer	Health economics	- INSERM, UMR912 (SESSTIM), Marseille, France<br>- Aix Marseille Université, UMR_S912, IRD, Marseille, France<br>- ORS PACA, Observatoire Régional de la Santé Provence-Alpes-Côte d'Azur, Marseille, France
Alexandra Calmy	Adult Medicine	- Service des maladies infectieuses, Hôpital Universitaire de Geneve, Genève.
Vincent Calvez	Virology	- Department of virology, Hôpital Pitié-Salpétrière, Paris, France
Marie-Laure Chaix	Virology	- EA 3620, Université Paris-Descartes, Laboratoire de Virologie, Hôpital Necker-Enfants Malades, AP-HP, Paris
Rosemary Dray-Spira	Social sciences	- INSERM U1018, CESP, Epidemiology of Occupational and Social Determinants of Health, Villejuif, France

		- University of Versailles Saint-Quentin, UMRS 1018, Villejuif, France
Kamal ElFarouki	Social sciences	- INSERM U1018, CESP, Epidemiology of Occupational and Social Determinants of Health, Villejuif, France
		- University of Versailles Saint-Quentin, UMRS 1018, Villejuif, France
Kenneth Freedberg	Modelling	- Massachusetts General Hospital, Harvard Medical School, Boston, MA, USA.
Kobus Herbst	Data management	- Africa Centre for Health and Population Studies, University of KwaZulu-Natal, South Africa
John Imrie	Social sciences	- Futures Group, Johannesburg, South Africa
		- Centre for Sexual Health and HIV Research, Research Department of Infection and Population, Faculty of Population Health Sciences, University College London, London, UK
Sophie Karcher	Data management	- Univ. Bordeaux, ISPED, Centre Inserm U897- Epidemiologie-Biostatistique, Bordeaux, France
		- INSERM, ISPED, Centre Inserm U897- Epidemiologie-Biostatistique, Bordeaux, France
Joseph Larmarange	Social sciences	- CEPED (Centre Population & Développement-UMR 196-Paris Descartes/INED/IRD), IRD (Institut de Recherche pour le Développement), Paris, France.
		- Africa Centre for Health and Population Studies, University of KwaZulu-Natal, South Africa
France Lert	Social Sciences	- INSERM U1018, CESP, Epidemiology of Occupational and Social Determinants of Health, Villejuif, France
		- University of Versailles Saint-Quentin, UMRS 1018, Villejuif, France
Richard Lessells	Adult medicine	- London School of Hygiene and Tropical Medicine, UK
Thembisa Makowa	Field operations	- Africa Centre for Health and Population Studies, University of KwaZulu-Natal, South Africa
Anne-Geneviève Marcelin	Virology	- Department of virology, Hôpital Pitié-Salpétrière, Paris, France
Laura March	Health economics	- INSERM, UMR912 (SESSTIM), Marseille, France
		- Aix Marseille Université, UMR_S912, IRD, Marseille, France
		- ORS PACA, Observatoire Régional de la Santé Provence-Alpes-Côte d'Azur, Marseille, France
Nuala McGrath	Epidemiology/Social sciences	- Academic Unit of Primary Care and Population Sciences, and Department of Social statistics and Demography, University of Southampton, United Kingdom
Kevi Naidu	Adult medicine	- Africa Centre for Health and Population Studies, University of KwaZulu-Natal, South Africa
Colin Newell	Data management	- Africa Centre for Health and Population Studies, University of KwaZulu-Natal, South Africa
Nonhlanhla Okesola	Nurse manager	- Africa Centre for Health and Population Studies, University of KwaZulu-Natal, South Africa
Tulio de Oliveira	Bioinformatics	- Africa Centre for Health and Population Studies, University of KwaZulu-Natal, South Africa
Melanie Plazy	Epidemiology/social sciences	- Univ. Bordeaux, ISPED, Centre Inserm U897- Epidemiologie-Biostatistique, Bordeaux, France

		- INSERM, ISPED, Centre Inserm U897- Epidemiologie-Biostatistique, Bordeaux, France
Tamsen Rochat	Anthropology/psychology	- Africa Centre for Health and Population Studies, University of KwaZulu-Natal, South Africa
Bruno Spire	Health economics	- INSERM, UMR912 (SESSTIM), 13006, Marseille, France
		- Aix Marseille Université, UMR_S912, IRD, Marseille, France
		- ORS PACA, Observatoire Régional de la Santé Provence-Alpes-Côte d'Azur, Marseille, France
Frank Tanser	Epidemiology and Biostatistics	- Africa Centre for Health and Population Studies, University of KwaZulu-Natal, South Africa
Rodolphe Thiébaut	Epidemiology and Biostatistics	- Univ. Bordeaux, ISPED, Centre Inserm U897- Epidemiologie-Biostatistique, Bordeaux, France
		- INSERM, ISPED, Centre Inserm U897- Epidemiologie-Biostatistique, Bordeaux, France
Johannes Viljoen	Virology	- Africa Centre for Health and Population Studies, University of KwaZulu-Natal, South Africa
Thembelile Zuma	Psychology/Social sciences	- Africa Centre for Health and Population Studies, University of KwaZulu-Natal, South Africa

Scientific advisory board

- Chair: Bernard Hirschel (Switzerland)
- International experts: Xavier Anglaret (Ivory Coast), Hoosen Cooavdia (South Africa), Alpha Diallo (France), Bruno Giraudeau (France), Jean-Michel Molina (France), Lynn Morris (South Africa), François Venter (South Africa), Sibongile Zungu (South Africa)
- Community representatives: Eric Fleutelot (France), Eric Goemaere (South Africa), Calice Talom (Cameroon)
- Sponsor representatives (ANRS): Brigitte Bazin, Claire Rekacewicz
- Pharmaceutical company representatives: Golriz Pahlavan-Grumel (MSD), Alice Jacob (Gilead)

Data safety and monitoring board

- Chair: Patrick Yeni (France)
- Members: Sinead Delany-Moretlwe (South Africa), Nathan Ford (South Africa), Catherine Hankins (Netherlands), Helen Weiss (UK)
